# Supplementary material for: Rules of hierarchical melt and coordinate bond to design crystallization in doped phase change materials
Source: Nat Commun. 2021 Nov 9;12:6473. doi: 10.1038/s41467-021-26696-9 (PMC8578292; doi:10.1038/s41467-021-26696-9)
Supplement: Supplementary file 1 — Supplementary Information [file 41467_2021_26696_MOESM1_ESM.pdf]

Supplementary Information for

Rules of hierarchical melt and coordinate bond to design crystallization in doped phase change materials

Jin Zhao[1,2,3], Wen-Xiong Song[1*], Tianjiao Xin[1], Zhitang Song[1*]

[1]State Key Laboratory of Functional Materials for Informatics, Shanghai Institute of Microsystem and Information, Chinese Academy of Sciences, Shanghai 200050, China; [2]School of Physical Science and Technology, Shanghai Tech University, Shanghai 201210, China; [3]University of Chinese Academy of Sciences, Beijing, 100049, China. Corresponding author E-mail: songwx@mail.sim.ac.cn; ztsong@mail.sim.ac.cn.

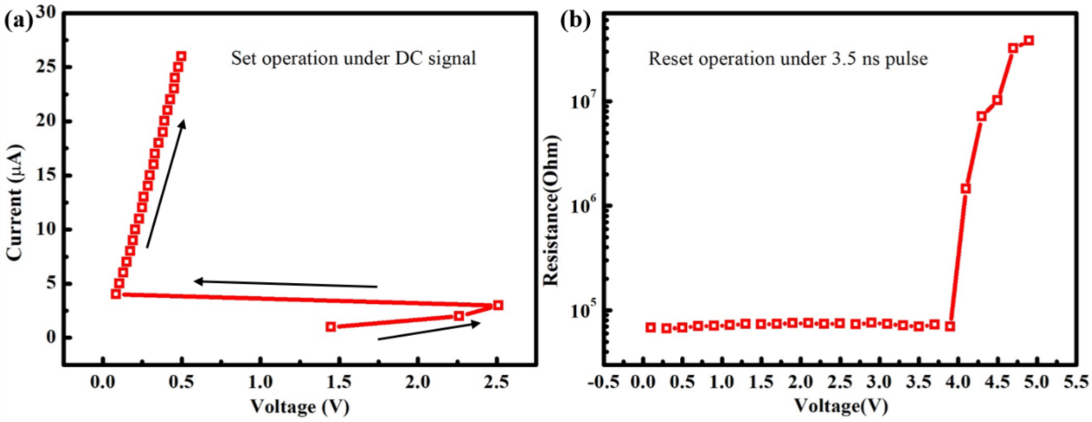

Supplementary Fig. 1 The initial special electrical operation to achieve hierarchical melt. **(a)**, applying a direct current signal to the cell, in which the cell resistance changes from the initial high-resistance amorphous state to the low-resistance crystalline state. **(b)**, less heat is provided by using the voltage pulse of 3.5 ns width and the magnitude ranging from 0.2 to 5.2 V, where the cell changes from the crystalline state to the high-resistance amorphous state. In our experiment, the SET operation on the as-deposited amorphous state is always hard to obtain high speed. We always firstly execute a SET operation with the direct current signal to obtain the crystalline state, as shown in (a). Then, the cell is programmed to a RESET state using an extremely narrow pulse signal, such as 3.5 ns herein, as shown in (b). According the hierarchical melt model proposed in the main text, the slowing down of cooperative movements by dopants with strong bonds prohibit the nucleation. The narrow pulse signal used in (b) helps obtain a medium-range ordered region, acting as a precursor accelerate the crystallization, because the wide pulse is hard to obtain this state.

Supplementary Table 1 Mismatch table for many tellurides (X_2Te_3) Tellurides formed by replacing Sb atoms with other atoms undergo structure relaxation. The atomic volume, lattice constant, and bond length mismatch rates are shown in Table.

Atomic number	Element	Average atomic volume ratio (%)	Lattice constant ratio in a- axis (%)	Lattice constant ratio in c-axis (%)	Bond length ratio (%)
13	Al	0.86967	7.36732	1.33651	8.08485
14	Si	1.26232	9.87052	0.20685	9.85018
21	Sc	0.58167	4.59593	0.28074	5.96787
22	Ti	1.51968	11.0994	2.31344	9.91803
23	V	2.09897	17.64116	1.50226	12.22132
24	Cr	2.17619	21.48509	11.00027	13.71504
25	Mn	2.37205	21.46618	4.44849	14.73796
26	Fe	2.57368	15.04249	14.93957	15.64138
27	Co	2.60197	14.99591	15.62	15.78856
28	Ni	2.44559	12.198	17.86927	15.18524
29	Cu	2.2008	8.01324	20.83269	12.03186
30	Zn	1.67548	6.56413	14.24338	8.02827
39	Y	0.4064	1.19222	3.61067	6.62475
40	Zr	0.81558	5.68875	1.0998	6.1941
41	Nb	1.83322	17.92872	7.63806	8.00681
42	Mo	1.88099	21.39192	16.85792	10.30989
43	Tc	2.12516	21.67764	11.0503	11.65115
44	Ru	2.07403	13.96211	6.93769	12.08246
45	Rh	2.08117	11.51333	12.15409	12.31458
46	Pd	1.956	6.44167	19.27489	10.62272
47	Ag	1.52979	3.70003	16.91206	6.27407
57	La	1.39227	7.71461	4.18855	7.23716
58	Ce	0.74724	3.64884	3.51654	5.1611
59	Pr	1.14927	6.00722	4.32814	6.88619
60	Nd	0.98783	5.17705	3.7925	6.74137
61	Pm	0.92286	4.02172	5.21021	2.17974
62	Sm	0.79511	3.35113	4.78599	6.70719
63	Eu	0.42887	2.97409	0.37381	4.96222
64	Gd	0.46122	1.9313	2.9051	6.67046
65	Tb	0.47503	1.61861	3.73995	6.57781
66	Dy	0.33316	0.94271	3.04542	6.59383
67	Ho	0.27894	0.48773	3.17521	6.62997
68	Er	0.01822	0.755	1.76232	2.45138
69	Tm	0.19825	0.05212	3.08112	6.68853
71	Lu	0.01604	0.85047	1.4782	6.40657
72	Hf	0.78354	5.80383	1.0386	7.05003
73	Ta	1.63227	19.38867	15.98341	8.1879
74	W	1.94082	22.45454	18.1846	9.83957
75	Re	2.12855	23.29601	15.69941	10.99302
76	Os	1.94733	11.98884	8.61033	12.06583
77	Ir	2.05571	9.1704	16.1645	12.23356
78	Pt	1.91552	7.02695	17.55292	10.83462
79	Au	1.59007	3.63615	17.99594	7.28907
83	Bi	0.48292	2.5198	2.03675	2.36692

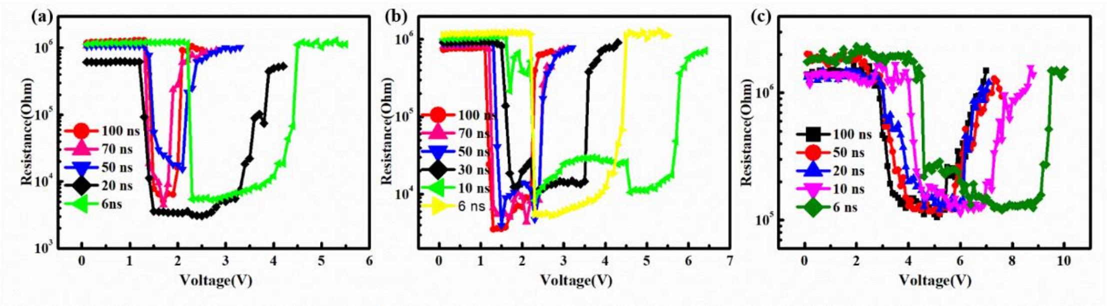

Supplementary Fig. 2 The SET-RESET windows for **(a)** $Er_{0.31}Sb_2Te_3$, **(b)** $Er_{0.41}Sb_2Te_3$ and **(c)** Sb_2Te_3 cell devices. The RESET voltages corresponding to the fast SET speed of 6 ns are 4.5 V, 6.1 V and 9.7 V. Figures show the device performance of the $Er_{0.31}Sb_2Te_3$ and $Er_{0.41}Sb_2Te_3$ system, both of which has the fastest speed of 6 ns. For comparison, the pure Sb_2Te_3 is shown in Fig. S3c. We also test the performance of the $Er_{0.76}Sb_2Te_3$ component, but does not have phase-change characteristics. It is noted that the $Er_{0.52}Sb_2Te_3$ component in the main text has the fastest speed of 3.2ns.

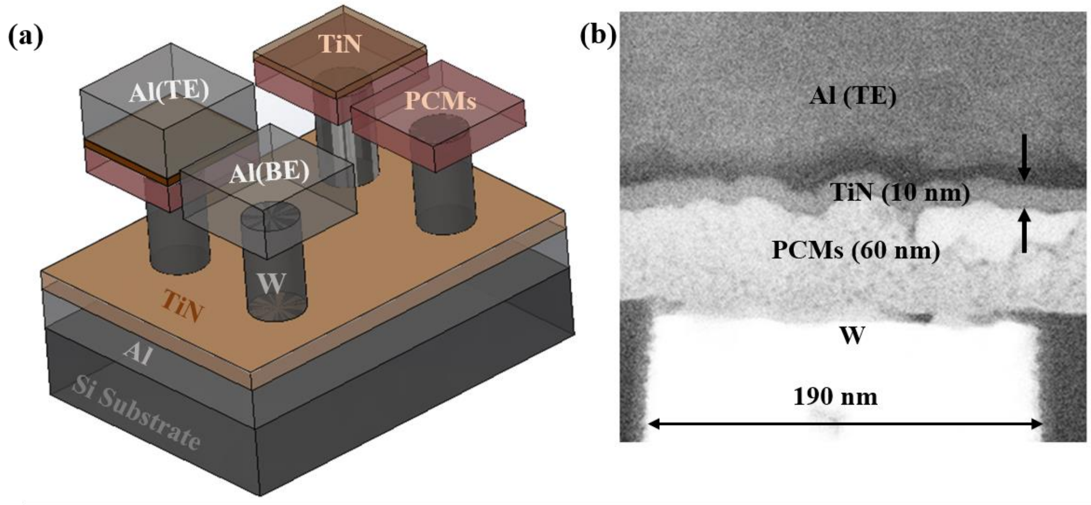

Supplementary Fig. 3 Device structure. **(a),** schematic diagram of T-type phase change memory cell. **(b),** the TEM image of the cross-section of the memory cell. T-shaped phase-change random-access memory (PCRAM) devices with tungsten plug bottom electrode contact (BEC, diameter = 190 nm) are fabricated using the 0.13 μm node complementary metal-oxide semiconductor technology.

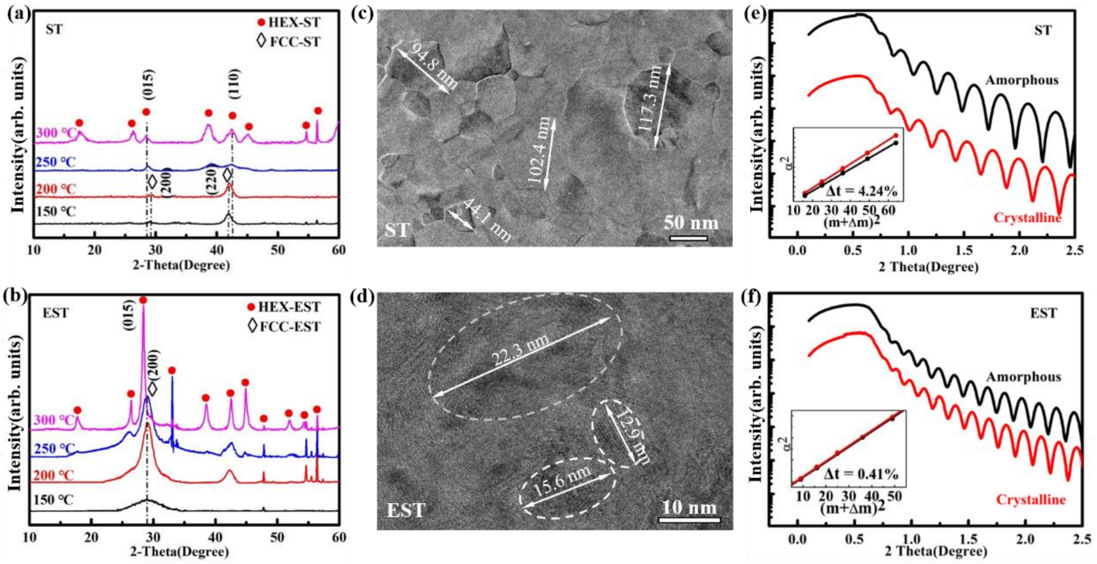

Supplementary Fig. 4 The structure change of the ST and EST with the evolution of temperature. **(a-b)**, XRD patterns of Sb_2Te_3 and $Er_{0.52}Sb_2Te_3$ films at different annealing temperatures. Both ST and EST samples appear diffraction peaks of face-centered cubic (*f*-) phase, which can be confirmed by the (200) and (220) peaks (marked by rhombus) at low temperature 150 °C and 200 °C. As the temperature increases to 250 °C, the peaks of *f*-phase disappear completely in Sb_2Te_3 whereas the (200) peak of *f*-phase across the EST still exists. The (200) peak of EST disappears until the temperature reaches 300 °C and the (015) peak of hexagonal (*h*-) phase appears on the curve. **(c-d)**, TEM images of the crystalline Sb_2Te_3 (230 °C) and $Er_{0.52}Sb_2Te_3$ (280 °C) films. The grain size of ST is as large as ~100 nm, while it is just a dozen nanometers in EST. **(e-f)**, The density-change rate between the crystalline and amorphous phases characterized by the XRR curves in the Sb_2Te_3 and $Er_{0.52}Sb_2Te_3$ films. The inset shows Bragg fitting curves of amorphous and crystalline films, and it shows that Er-doping results in the thickness change decreased from 4.24 % to 0.41 %. The XRD experiment reveals that doping Er can increase the temperature of the *f*-to-*h* phase transition from 250 °C to 300 °C, which is similar to the transition temperature of GST. The metastable EST phase can be promising for realizing reversibly and rapidly swift and low-energy PCRAM applications, while the occurrence of hexagonal phase probably fails the device[1]. The TEM images show that Er dopant can refine the grains seriously, and grain refinement can reduce power consumption via boundary scattering phonons to reduce heat loss, which has been systematically studied in the previous study.[2] Accompanied by significant grain refinement, the resulting more stable grain boundaries suppress the internal stress of the material, and the resulting EST has a ultra-low density-change rate of 0.41 %, which is much lower than that of $Ge_2Sb_2Te_5$ (6.5%)[3].

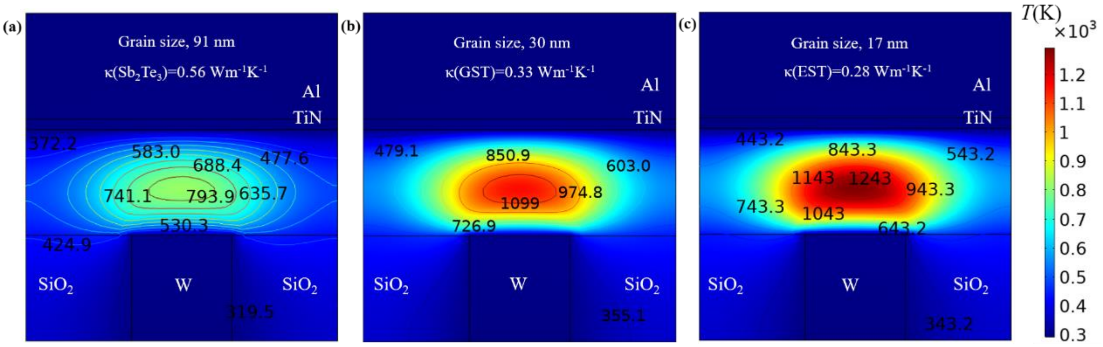

Supplementary Fig. 5 Finite element analysis of low power consumption **(a)** Sb_2Te_3, **(b)** GST, and **(c)** EST are simulated by the finite element method with different thermal conductivity. The working area of the EST device has higher temperature using the same heat source, whose thermal conductivity value is the smallest compared with the other two materials. The finite element method (FEM) is used to analyze the temperature distribution based on ST, GST and EST. We estimated the thermal conductivity according the previous proposed formula according to the grain size[2], $\kappa = L_d D + \kappa_0$, where D is the average diameter of grain size, L_d is the coefficient estimated 3.67×10^6 $Wm^{-2}K^{-1}$, and κ_o (0.22 $Wm^{-1}K^{-1}$ herein) is the thermal conductivity of amorphous. We find that EST of the less grain size has more energy efficient than other two models, because more grain boundaries scatter the phonons.

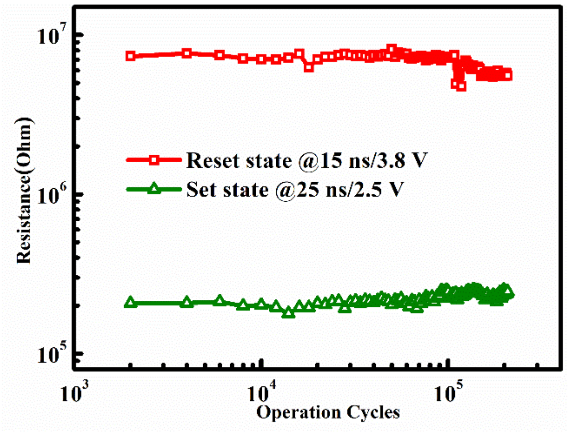

Supplementary Fig. 6 The operation cycles of $Er_{0.52}Sb_2Te_3$ under the SET of 25ns/2.5V and RESET of 15ns/3.8V.

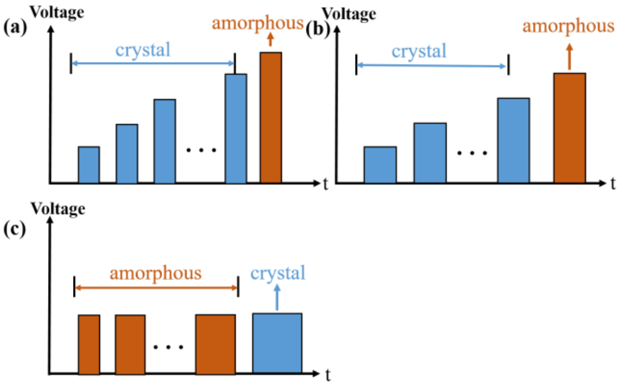

Supplementary Fig. 7 Different pulse waveforms applied to the PCRAM devices. **(a-b)**, iterative RESET operations with different pulse width to obtain the amorphous state. **(a)** and **(b)** always obtain the short and long melting time sates, respectively. **(c)**, SET operations. The iterative SET pulse waveforms with the constant voltage are used to measure the SET speed. We use the iterative RESET operation to obtain the different melting states. We employ the incremental voltage pulses (0.1V herein) at a constant width, such as 100 ns in (a) and 1000 ns in (b). Until the final pulse (brown) imposed, the previous pulses (blue) cannot melt the cell. Thus, the last critical pulse with different width and voltage value provides the different melting states. Subsequently, we immediately apply the SET pulses with gradually increased pulse width to test the SET speed, as shown in (c). The "SET Speed" is defined as a critical pulse width after the sudden resistance reduce since the crystallization.

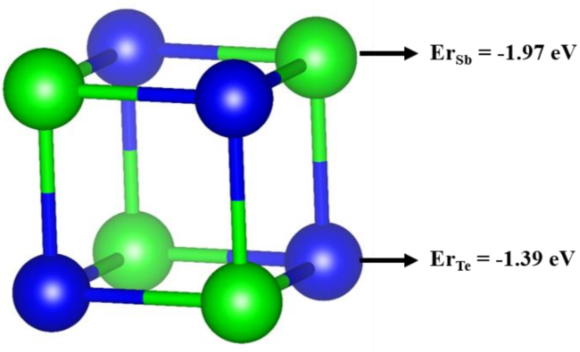

Supplementary Fig. 8 Two representative doping sites in the crystalline Sb_2T_3 and the corresponding substitution energy. The *ab-initio* method is used to predict the most stable site by calculating the substitution energy (E_s). The formation energy (E_f) can be calculated by the formula[4], $E_f = (E_{\mathrm{EST}} + E_{\mathrm{Sb/Te}}) - (E_{\mathrm{ST}} + E_{\mathrm{Er}})$, where E_{EST} and E_{ST} represent the total energy of the relaxed structure with and without Er atom, respectively. $E_{\mathrm{Sb/Te}}$ is the chemical potential of Sb or Te atoms, and E_{Er} is the chemical potential of Er atom. Er_{Sb} (-1.97 eV) has a lower E_f value than Er_{Te} (-1.39 eV), which illustrates that the preferred position for Er is Sb position.

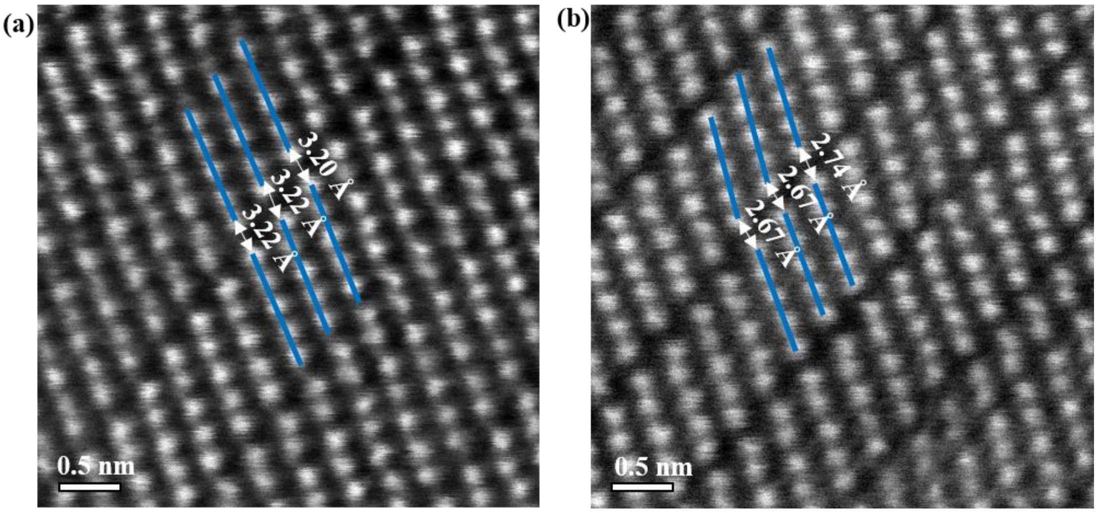

Supplementary Fig. 9 The HAADF image of the crystalline EST film structure. **(a)**, the cubic phase of EST. **(b)**, the hexagonal phase of EST. The positions marked are the atomic distance at the Te-Te gaps. The cubic phase presents a continuous atomic network along the [110] direction, even the randomly distributed vacancies can aggregate into a layer shape, as shown in (a). While the hexagonal phase has the quintuple-layered building blocks separated by Te···Te van der Waals gaps, the Te···Te van der Waals gap is smaller than vacancy layer. For the Te···Te gap, the vacancy layer of ~3.2Å in the cubic structure is wider than van der Waals layer (2.7 Å) in the hexagonal phase.

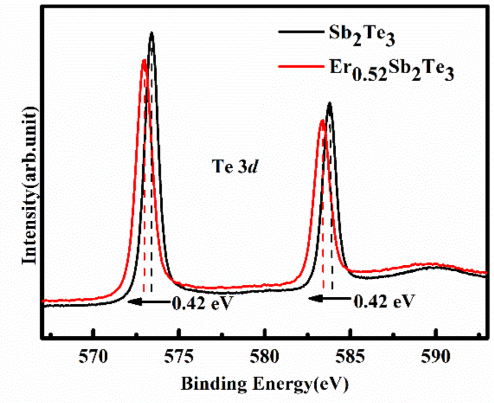

Supplementary Fig. 10 XPS spectra for annealed Sb_2Te_3 and $Er_{0.52}Sb_2Te_3$ films. The binding energy of Te 3*d* orbital presents 0.42 eV redshift. XPS experiment is applied to investigate the bonding state of crystallized EST and ST films. The binding energy of the Te 3*d* can be identified, and the redshift of binding energy of them is clearly visible after doping Er.

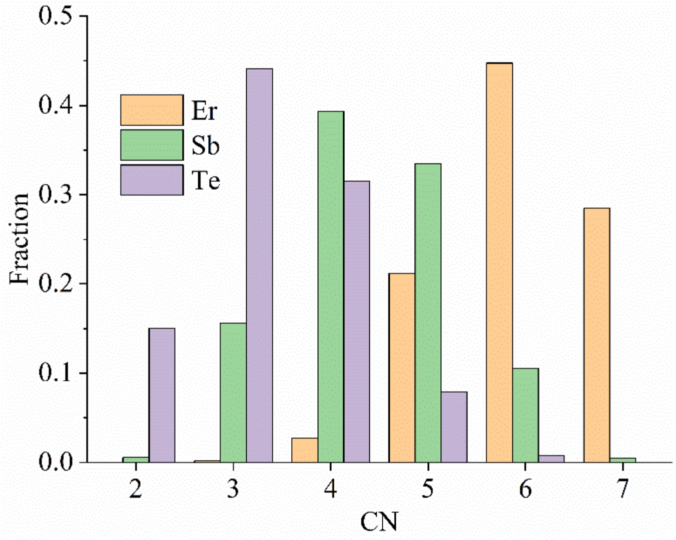

Supplementary Fig. 11 The statistics of CN for Er, Sb, and Te atoms in the amorphous. The CN of Er broadly distribute around 6, while CNs for Te are mainly 3~4, and CNs for Sb are mainly 4~5. The used cutoff is 3.4 Å. The local structural information around Er, Sb and Te atoms in amorphous Er doped Sb_2Te_3 by AIMD simulations. Er has the largest average coordination number (CN) of about six.

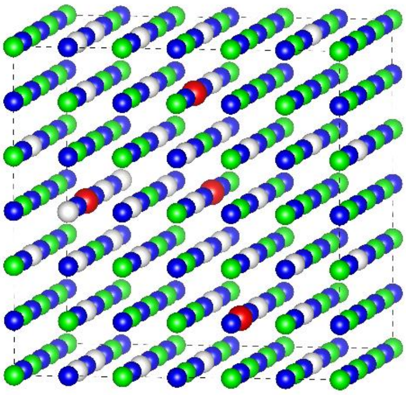

Supplementary Fig. 12 A 6 × 6 × 6 rock-salt supercell model. Vacancy, Er, Sb, and Te atoms are marked by white, red, green, and blue, respectively. Instead of using a random vacancy model, planar ordering vacancies are placed in the 6 × 6 × 6 rock-salt supercell model and Er atoms near the vacancies, as shown in Fig. S13. It is because Er atom prefers the cationic position near vacancies from the experiment and calculation results in the main text. This model is melted to obtain a medium-range region stabilized by Er atoms, which acts as a precursor to accelerate crystallization.

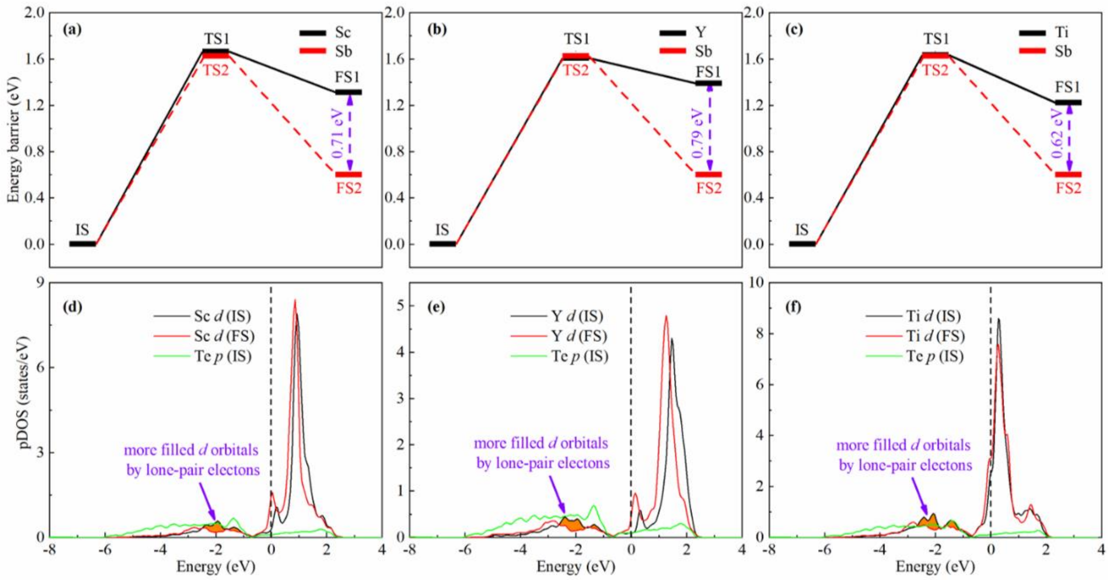

Supplementary Fig. 13 Stabilized Sc, Y, and Ti with empty d orbitals filled by lone-pair electrons (a-c), the energetic profile of diffusion barrier for Sc, Y, Ti (black) or Sb (red and dash), where a model with a four-vacancy-aggregated cluster in the (111) plane is shown in the inner graph of Fig. 4e. **(d-f)**, the partial DOS of Sc, Y, Ti *d* (black) and Te *p* (green) orbitals in the IS structure, while the Sc, Y, Ti *d* (red) orbitals in the FS structure also shown for comparison. It is noted that the pDOS of Te *p* orbitals is divided by Te number (48 herein). The same diffusion model used in main text of Fig. 4e, the diffusion barrier in Sc-Sb_2Te_3, Y-Sb_2Te_3, and Ti-Sb_2Te_3 systems is calculated, as shown in (a-c). The much higher energy of FS states illustrates that Sc, Y and Ti atoms prefer the IS positions that are near vacancies. It is because the less lone-pair electrons shared with the empty *d* orbitals as dopant atoms in the FS states, which results in the relative energy of 0.71 eV for Sc, 0.79 eV for Y, and 0.62 eV for Ti higher than the reference model of replacing the corresponding dopants by a Sb atom.

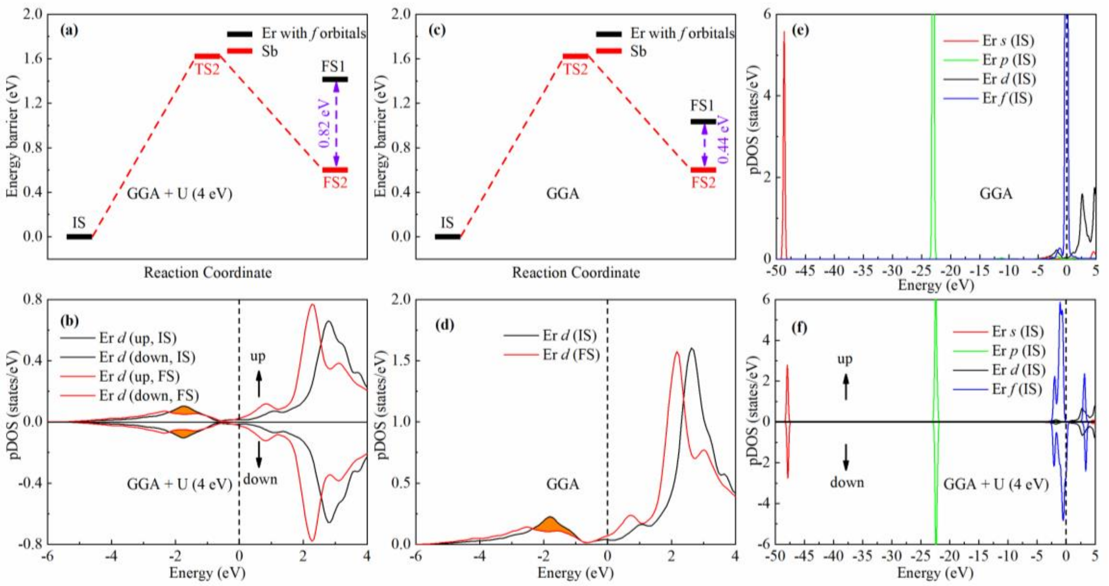

Supplementary Fig. 14 The effect of Er *f* orbitals **(a)-(b)**, the relative energy **(a)** and partial DOS **(b)** of the IS and FS1 structures, when the Hubbard *U* value of 4 eV is considered for Er *f* orbitals. **(c)-(d),** the relative energy **(c)** and partial DOS **(d)** of the IS and FS structures, without Hubbard *U* value considered for Er *f* orbitals. **(e)-(f),** the partial DOS of Er *s*, *p*, *d*, and *f* orbitals with Hubbard *U* value **(f)** or not **(e)**. The valence electrons in this figure are Er $5p^6 5d^1 6s^2$, Sb $5s^2 5p^3$, Te $5s^2 5p^4$. In order to investigate the effect of Er 4*f* orbitals on the calculation results in the main text, we consider Er 4*f* electrons as valence electrons to calculate the diffusion barrier of Er migration, where the same model is used in Fig. 4. Figures shows the relative energy and partial DOSs of the IS and FS1 structures. The relative energy of 0.82 eV is similar to the value shown in Fig. 4e of 0.75 eV, where the Hubbard *U* with 4 eV is used to correct Er 4*f* electrons localization. It is noted that the suggested Hubbard *U* value is 6.86 eV,[5] but it is hard to be converged herein. Without Hubbard *U* value added, the relative energy difference of FS1 and FS2 is 0.44, about half value of 0.75 eV. Although the two scenarios give different relative values for the FS1 and FS2 structures, they still predict that the Er atoms in FS1 structure is unstable. It is because the less lone-pair electrons fill the empty Er 5*d* orbitals, as shown in Fig. S15b,d. Finally, we calculate the partial DOS of Er *s*, *p*, *d*, and *f* orbitals, as shown in (e,f). Although the energy of the 4*f* orbitals is close to the Fermi level, it is in fact the case that *f*-electrons do not play a major role in the bonding of rare-earth compounds.[6, 7] It demonstrates that the pseudopotential performs well as the Er 4*f* electrons included in the core.

Supplementary References

1. Gao D, Liu B, Xu Z, Li Y, Wang L, Song Z*, et al.* Failure Analysis of Nitrogen-Doped Ge2Sb2Te5Phase Change Memory. *IEEE T. Device Mat. Re.* **16,** 74-79 (2016).
2. Song W-X, Cheng Y, Cai D, Tang Q, Song Z, Wang L*, et al.* Improving the performance of phase-change memory by grain refinement. *J. Appl. Phys* **128,** 075101 (2020).
3. Njoroge WK, Woltgens HW, Wuttig M. Density changes upon crystallization of Ge2Sb2.04Te4.74 films. *J. Vac. Sci. Technol. , A* **20,** 230-233 (2002).
4. Zhang S, Northrup J. Chemical potential dependence of defect formation energies in GaAs: Application to Ga self-diffusion. *Phys. Rev. Lett* **67,** 2339-2342 (1991).
5. Sanna S, Hourahine B, Frauenheim T, Gerstmann U. Theoretical study of rare earth point defects in GaN. *phys. status solidi C* **5,** 2358-2360 (2008).
6. Sanna S, Hourahine B, Gerstmann U, Frauenheim T. Efficient tight-binding approach for the study of strongly correlated systems. *Phys. Rev. B* **76,** (2007).
7. O'Donnell K, Dierolf V. *Rare Earth Doped III-Nitrides for Optoelectronic and Spintronic Applications*, vol. 124, 2010.
